# High Polygenic Risk Scores Positively Associated with Gastric Cancer Risk Interact with Coffee and Polyphenol Intake and Smoking Status in Korean Adults

**DOI:** 10.3390/nu16193263

**Published:** 2024-09-27

**Authors:** Meiling Liu, Sang-Shin Song, Sunmin Park

**Affiliations:** 1 Department of Chemical Engineering, Shanxi Institute of Science and Technology, Jincheng 048000, China; liumeiling@sxist.edu.cn; 2 Department of Food and Nutrition, Obesity/Diabetes Research Center, Hoseo University, Asan 31499, Republic of Korea; ssscho@empas.com

**Keywords:** gastric cancer, white blood cells, polygenic variants, coffee, smoking status, polyphenols

## Abstract

Background/Objectives: This study investigated the relationship between single nucleotide polymorphisms (SNPs) and gastric cancer (GC) risk, while also examining the interaction of genetic factors with lifestyle variables including the nutrient and bioactive compound intake in Korean adults of a large hospital-based cohort. Methods: We conducted a genome-wide association study (GWAS) comparing GC patients (*n* = 312) with healthy controls without cancers (*n* = 47,994) to identify relevant genetic variants. Generalized multifactor dimensionality reduction (GMDR) was employed to detect SNP interactions between diets and lifestyles. We utilized polygenic risk scores (PRSs) to assess individuals’ GC risk based on multiple SNP loci. Among the selected SNPs, since *SEMA3C*_rs1527482 was a missense mutation, bioactive compounds which decrease the binding energy were found with its wild and mutated proteins by molecular docking analysis. Results: Individuals with high PRSs exhibited a 4.12-fold increased risk of GC compared to those with low PRSs. Additional factors associated with elevated GC risk included a low white blood cell count (OR = 5.13), smoking (OR = 3.83), and low coffee consumption (OR = 6.30). The *SEMA3C*_rs1527482 variant showed a positive correlation with GC risk. Molecular docking analyses suggested that certain polyphenols, including theaflavate, rugosin E, vitisifuran B, and plantacyanin, reduced the binding free energy in both wild-type and mutated *SEMA3C*_rs1527482. However, some polyphenols exhibited differential binding energies between its wild and mutated forms, suggesting they might modulate wild and mutated proteins differently. Conclusion: High PRSs and *SEMA3C*_rs1527482 interact with immune function, coffee intake, polyphenol consumption, and smoking status to influence GC risk. These findings could contribute to developing personalized nutrition and lifestyle interventions to reduce GC risk.

## 1. Introduction

Gastric cancer remains a significant global health concern, ranking as the fifth most common cancer worldwide (5.6%) according to 2020 estimates from the International Agency for Research on Cancer (IARC) [1]. Its mortality rate ranks fourth (7.7%) among cancers globally [1]. In Korea, the gastric cancer incidence in men was second only to thyroid cancer as of 2018. The disease primarily manifests as adenocarcinomas, originating from the stomach’s glandular cells and accounting for 95% of cases, with a notably higher prevalence in men [2].

The etiology of gastric cancer is multifaceted, involving complex interactions between bacterial infections, environmental factors, and genetic predisposition. Modifiable risk factors, including lifestyle and dietary habits, are crucial in gastric cancer development [3]. Geographic variations in the disease’s presentation suggest that the interplay between environmental and genetic factors may differ across populations.

Previous genetic studies have identified several single-nucleotide polymorphisms (SNPs) associated with gastric cancer risk. These include variants in tumor suppressor genes (cadherin 1 [*CDH1*], tumor protein 53 [*TP53*]) [4,5], genes involved in mucosal protection against *Helicobacter pylori* (*H. pylori*) (interleukin-1 beta [*IL1B*], interleukin-1 receptor antagonist protein [*IL1RN*], and tumor necrosis factor-α [*TNF-α*]) [6,7], carcinogen metabolism genes (cytochrome P450 family 2 subfamily E member 1 [*CYP2E1*], glutathione S-transferase mu 1 [*GSTM1*]) [8,9], and those related to DNA repair (methylenetetrahydrofolate reductase [*MTHFR*] and X-ray repair cross-complementing group 1 gene [*XRCC1*]) [10,11]. Recent research in Korean populations has uncovered additional genetic markers, including protein kinase AMP-activated catalytic subunit alpha 1 (*PRKAA1*) [12], mucin 1 (*MUC1)*, phospholipase C epsilon 1 (*PLCE1*) [13], and prostate stem cell antigen (*PSCA*) [14].

While numerous studies have explored the relationship between dietary factors, environmental influences, and gastric cancer risk, the results have often been inconclusive. Moreover, genetic investigations have typically focused on individual susceptibility genes rather than considering the cumulative effect of multiple genetic variants. The concept of polygenic risk scores (PRSs) offers a more comprehensive approach to assessing genetic predisposition to gastric cancer.

Limited research has examined the interaction between dietary patterns and combinations of genetic variants in identifying high-risk groups for gastric cancer. Previous studies have analyzed the association between PRSs and the age at onset of gastric cancer [15] and predicted gastric cancer risk using PRSs according to *H. pylori* infection status [16]. A meta-analysis has also suggested that a healthy lifestyle can mitigate the genetic risk of gastric cancer [17].

This study aimed to address this gap by investigating the hypothesis that polygenic variants interact with metabolic parameters, dietary intake, and lifestyle factors to influence gastric cancer risk. Utilizing data from the Korean Genome and Epidemiology Study (KoGES), we evaluated the interplay between PRSs for gastric cancer and various factors, including immune function, lifestyle choices, and environmental exposures in the Korean adult population. Our findings suggested significant interactions between genetic predisposition and modifiable risk factors, highlighting potential avenues for targeted interventions to mitigate the gastric cancer risk in genetically susceptible individuals. These results underscored the need for further research to validate and expand upon these observations, potentially informing future strategies for early prevention and risk reduction in high-risk populations.

## 2. Methods

### 2.1. Study Population

This study utilized data from the Korea Genomic and Epidemiological Study (KoGES), a large urban hospital cohort study conducted by the Korea Disease Control and Prevention Agency (KDCA) between 2004 and 2013. The cohort included Korean adults aged ≥40 years (*n* = 58,701) who volunteered to participate. The KoGES protocol was approved by the institutional review boards of the Korean National Institute of Health (Approval Code: KBP-2015-055, approved on 20 August 2015), and all participants provided written informed consent.

### 2.2. Case-Control Selection

**Figure 1:**
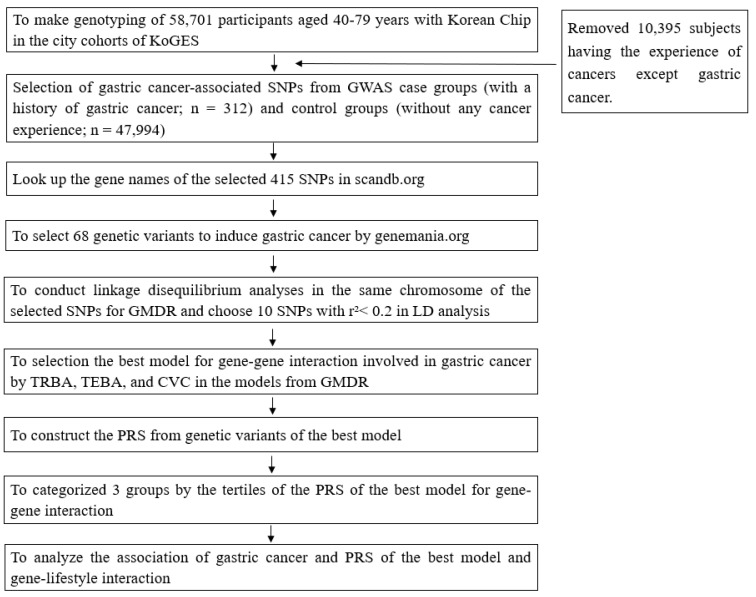
Flow chart for the generation of polygenic risk scores (PRSs) that influence gastric cancer risk and their interaction with metabolic parameters and lifestyles. KoGES, Korean Genome and Epidemiology Study; SNP, single nucleotide polymorphism; GWAS, genome-wide association study; LD, linkage disequilibrium; GMDR, generalized multifactor dimensionality reduction; TRBA, trained balanced accuracy; TEBA, test balance accuracy; CVC, cross-validation consistency; PRS, polygenic risk scores.

Participants were categorized into two groups based on their self-reported gastric cancer diagnosis. The gastric cancer group (GC, cases; *n* = 312) comprised individuals who reported a physician-diagnosed gastric cancer, and subjects with any history of cancer other than gastric cancer were excluded (*n* = 10,395) (Figure 1). The non-gastric cancer group (N-GC, controls; *n* = 47,994) included participants without any reported cancer diagnoses. While different cancers might share some biological pathways or risk factors, their effects could act as a bias in determining gastric cancer risk. By excluding other cancers, we reduced the risk of these shared factors obscuring gastric cancer-specific genetic associations. This targeted approach allowed for a more precise investigation into the genetic basis and risk factors unique to gastric cancer, potentially leading to clearer and more robust results. Ultimately, this exclusion criterion helped to isolate the genetic factors specifically associated with gastric cancer, enabling a more accurate analysis of its pathogenesis.

### 2.3. Anthropometric and Biochemical Measurements

Demographic information, including age, income, education, alcohol consumption, smoking history, and physical activity, was collected through health interviews [18]. Education levels were categorized as less than high school, high school, and college or higher. Monthly household income was classified as low (<$2000), medium ($2000–4000), and high (>$4000). Smoking status was categorized as non-smoker, former smoker, or current smoker, while alcohol consumption was classified as light drinker (0–20 g/day) or moderate drinker (>20 g/day) [19].

Anthropometric measurements, including weight, height, and waist circumference, were taken by trained specialists using standardized procedures. Body mass index (BMI) was calculated as weight (kg) divided by height (m) squared. Blood samples were collected after a minimum 12 h fast to ensure accurate biochemical analysis [20]. A Hitachi 7600 automatic analyzer (Hitachi, Tokyo, Japan) was used to measure fasting glucose, serum total cholesterol, triglycerides, and high-density lipoprotein cholesterol (HDL-C) levels. White blood cell (WBC) counts were determined from ethylenediaminetetraacetic acid (EDTA)-treated blood samples. Blood pressure measurements were taken with participants in a seated position, with the right arm properly supported at heart level.

### 2.4. Dietary Assessment

Dietary intake was evaluated using a semiquantitative food frequency questionnaire (SQFFQ) developed and validated for the KoGES. The SQFFQ covered 106 food items, assessing long-term dietary patterns based on portion size and frequency of consumption. Nutrient intake was estimated using the computer-aided nutritional analysis program CAN-pro version 3.0, developed by the Korean Nutrition Society [21]. The dietary inflammatory index (DII) was calculated to assess the pro-inflammatory potential of participants’ diets. The index was computed using 38 food and nutrient components, excluding garlic, ginger, saffron, and turmeric due to a lack of intake data. DII scores were determined by multiplying component-specific inflammatory scores by daily intake, summing these products, and dividing by 100.

### 2.5. Genotyping and Quality Control

Genomic DNA extraction from whole blood and genotyping were performed by the Center for Genome Science at the National Institute of Health in Korea using a Korean-specific gene chip produced by Affymetrix (Santa Clara, CA, USA) [22]. Genotyping accuracy was determined using the Bayesian robust linear modeling with Mahalanobis distance genotyping algorithm [22]. Strict quality control parameters were applied to ensure data accuracy and representativeness, including genotyping accuracy (≥98%), genotype missing rate (<4%), heterozygosity (<30%), Hardy–Weinberg equilibrium (*p* > 0.05), and minor allele frequency (MAF > 0.01) [23].

### 2.6. Selection of Interacting Genetic Variants for Gastric Cancer

A polygenic risk score (PRS) for gastric cancer was developed using a multi-step process (Figure 1). Genome-wide association study (GWAS) methods were employed to identify genetic loci significantly associated with gastric cancer risk after adjusting for age, gender, region of residence, survey year, BMI, daily energy intake, education level, and income. The statistical significance was used for a more liberal cut-off of Bonferroni correction (*p* < 5 × 10^−4^) since there was a limited number of participants with gastric cancer. This approach allowed us to identify potentially important SNPs that might have been missed with a stricter threshold, while still accounting for multiple comparisons. From 415 initially identified variants, gene names were determined using scandb.org (accessed on 5 December 2021) and further screened using genemania.org. Linkage disequilibrium (LD) analysis was performed using Haploview 4.2 in the PLINK toolset to identify and exclude strongly linked SNPs (D′ < 0.4) among the selected 68 SNPs. Finally, 10 potential genetic variants from the best model and on the same chromosome were selected.

The best model for SNP–SNP interactions influencing gastric cancer risk was determined using generalized multifactor dimensionality reduction (GMDR) [24], based on trained balanced accuracy (TRBA), test balance accuracy (TEBA), and cross-validation consistency (CVC). The significance threshold was set at *p* < 0.001 to account for multiple tests. The number of risk alleles for each SNP in the best model was added to obtain the PRS for each individual. The calculated PRS was classified into tertiles, that is, the population was divided into three risk levels: low risk, medium risk, and high risk. A higher PRS value indicates that the individual has more risk alleles in the best gene interaction model and thus has a higher risk of gastric cancer.

### 2.7. Molecular Docking and Molecular Dynamics Simulation (MDS) of Semaphorin-3C (SEMA3C)

The I-TASSER website (https://zhanggroup.org/I-TASSER/ (accessed on 13 January 2023)) was used to predict the Protein Data Bank (PDB) structures of wild-type and mutant *SEMA3C* (Semaphorin 3C) proteins. The PDB format of *SEMA3C* protein and food components (*n* = 20,000) was converted to PDBQT files using AutoDock Tools 1.5.6 (Molecular Graphics Laboratory, Scripps Research Institute, Jupiter, FL, USA) [25]. The active sites, active functional pockets of SEMA3C, and mutated sites were found using the ProteinsPlus website (https://proteins.plus/ (accessed on 30 January 2023)) and included in molecular docking. Water molecules attached to the protein and food components were removed [25]. After docking was completed, the output binding energy estimates for each docked pose were analyzed, and those food components with binding energies less than −10.0 kcal/mol were selected as potential binding partners [26]. Binding affinity is a measure of the strength of binding between two molecules. It is usually inversely proportional to the binding free energy, that is, the lower the binding free energy, the higher the binding affinity, making the interaction between the two molecules more stable [26].

Molecular dynamics simulation (MDS) was used to study the conformational changes of protein structures after binding to specific food ingredients. After determining the optimal docking pose, molecular dynamics simulation was used to simulate the dynamic behavior of the complex after *SEMA3C* bound to the food ingredient. During the simulation, the Chemistry at Harvard Macromolecular Mechanics (CHARMM) force field was applied. In order to make the simulation closer to the real situation and more accurately reflect the behavior of the protein under physiological conditions, the protein was solvated, that is, placed in an environment of water molecules (or other solvents). The simulation was carried out for 10 nanoseconds (ns), and parameters such as the root mean square deviation (RMSD), root mean square fluctuation (RMSF), and hydrogen bond value were calculated during the simulation. These parameters are used to evaluate the stability of a protein conformation, the volatility of an atomic position, and the formation and breaking of intermolecular hydrogen bonds.

### 2.8. Statistical Analyses

Statistical analyses were performed using PLINK version 2.0 and SPSS version 24.0 (IBM SPSS Statistics, New York, NY, USA). SNP–SNP interactions were screened using GMDR, and the significance of SNP–SNP interactions was assessed by the signed-rank test of TRBA and TEBA. The best SNP–SNP interaction model with a *p*-value < 0.05 was selected. Covariates such as age, sex, BMI, region of residence, physical activity education, income level, smoking, drinking, and energy intake were adjusted or not [20]. Ten-fold cross-validation is a commonly used method to evaluate model performance, especially for cases with large sample sizes (*n* > 1000). This method ensures that every sample in the dataset has the opportunity to be used as a test set, thereby more comprehensively evaluating the generalization ability of the model and more accurately evaluating the reliability of the CVC [27]. Using the best model, as determined by GMDR analysis, the risk allele of each SNP in the selected best model was counted as 1 [28]. For example, when the G allele was associated with an increased risk of gastric cancer, the TT, GT, and GG were assigned scores of 0, 1, and 2. PRSs were calculated by summing the risk allele scores of each SNP. The best model with 8 SNPs was divided into three categories (0–6, 7–8, and ≥9) by tertile, that is, into low-, medium-, and high-PRS groups, respectively. Adjusted odds ratios (ORs) and 95% confidence interval (CI) for gastric cancer risk with PRS were calculated after adjusting for covariates. The covariates included were age, gender, BMI, region of residence, physical activity, education, income level, smoking, alcohol consumption, years with gastric cancer, and energy intake.

Descriptive statistical analyses were performed for categorical variables, such as sex and lifestyle, which were calculated based on the frequency distribution of the PRS tertiles (i.e., low-, medium-, and high-PRS groups). For the frequency distribution of categorical variables, the Chi-squared test was used for analysis. For quantitative variables, the Kolmogorov–Smirnov test was used to check their normality due to the large sample size, and was achieved by the proc univariate procedure. For variables that met the normal distribution, the means and standard errors were calculated according to the PRS tertile categories or the presence or absence of gastric cancer. To determine whether the differences were significant, a one-way analysis of variance (ANOVA) with covariance adjustment was used, and multiple comparisons between groups were performed using the Tukey test. In addition, to account for the interaction between PRSs and dietary intake parameters, participants were divided into high-intake and low-intake groups. After adjusting for covariates, two-way ANOVA with main effects and interaction terms was used to explore the interaction between PRSs and lifestyle parameters. Throughout the statistical analysis, a *p*-value of <0.05 was used as the criterion for statistical significance. This means that only when the observed differences reach or exceed this statistical significance level are they considered to be real and not due to random errors or sampling variations. Such statistical analysis methods help ensure the accuracy and reliability of research results.

## 3. Results

### 3.1. Comparison of the General Characteristics of the Participants

Table 1 describes the demographic and clinical characteristics of the participants, including 312 participants with gastric cancer and 47,994 without cancer. The average age of the GC group was 58 years, significantly higher than that of the N-GC group. The risk of gastric cancer in men was 3.37 times higher than in women (*p* < 0.001). The BMI (*p* < 0.001), plasma concentrations of total cholesterol and triglycerides (*p* < 0.01), and WBC counts (*p* < 0.05) were significantly lower in the GC group than in the N-GC group. The amounts of participants with high income (>$4000) and education levels (high school or > College) were also lower in the GC group than in the N-GC group (Table 1).

### 3.2. Comparison of Nutrient Intakes of the GC and N-GC Groups

Table 2 describes the nutrient intakes of the participants with and without gastric cancers. There was no difference in the intake of energy, carbohydrates, proteins, fats, sodium, and fiber between the GC and N-GC groups. The prevalence of previous smoking was higher in the GC group (*p* < 0.001), but the alcohol and coffee intake (*p* < 0.05) were lower than in the N-GC group. The incidence of gastric cancer may be possibly related to reduced alcohol and coffee consumption (Table 2). The total phenol intake was not significantly different between the N-GC and GC groups. However, the DII was lower in the GC group than in the N-GC group, indicating that the individuals experiencing GC had a better diet (Table 2).

### 3.3. Genetic Variants for Gastric Cancer Risk and the Best Model for Gene-Gene Interactions

The best gene variant–gene variant interaction model associated with gastric cancer risk was evaluated by the GMDR method. In order to select the best model among the 10 models shown in Table S1, statistical indicators such as the TRBA, TEBA, and CVC were tested. These indicators help to evaluate the predictive performance and stability of a model. When selecting the best model among the 10 models, the covariates listed in Table S1 were adjusted or not. By adjusting these covariates, the association between gene variants and gastric cancer risk can be more accurately evaluated and the influence of confounding factors can be reduced. The 10 selected SNPs were as follows: rs7521784 of disabled-1 (*DAB1*) on chromosome 1, rs12693006 of pyruvate dehydrogenase kinase-1 (*PDK1*) on chromosome 2, rs1045653 of dedicator-of-cytokinesis-10 (*DOCK10*) on chromosome 2, rs9835646 of zinc finger and BTB domain 20 (*ZBTB20*) on chromosome 3, rs630760 of Kalirin RhoGEF Kinase (*KALRN*) on chromosome 3, rs11946315 of a disintegrin and metalloprotease 29 (*ADAM29*) on chromosome4, rs1207808 of membrane-associated guanylate kinase 2 (*MAGI2*) on chromosome 7, rs58499534 of cub sushi multiple domains-1 (*CSMD1*) on chromosome 8, rs10831776 of molecule interacting with CasL-2 (*MICAL2*) on chromosome 11, and rs205881 of casein kinase IIA1 (*CSNK2A1*) on chromosome 20 (Table 3). Each genetic variant was significantly associated with gastric cancer (OR = 0.61–1.59; *p* = 1.70 × 10^−6^ to 0.0008587). The genotype frequency distribution met the HWE criteria (*p* > 0.05), and the minor allele frequency (MAF) value was *p* > 0.01 (Table 3).

This study listed multiple models in Table S1 and found that the eight-SNP model had the lowest *p*-value among all models. This suggested that the eight-SNP model was the best in revealing the association between SNP–SNP interaction and gastric cancer risk. In addition, the cross-validation consistency (CVC) value of the model was 10/10, which further confirmed the stability and reliability of the model. The CVC value reflects the prediction consistency of the model on different data subsets, and a high CVC value indicates that the model can maintain stable prediction performance under different conditions. As a result, this model, which included eight SNPs, including *DAB1*_rs7521784, *PDK1*_rs12693006, *DOCK10*_rs1045653, *MAGI2*_rs1207808, *CSMD1*_rs58499534, *MICAL2*_rs10831776, *CSNK2A1*_rs205881, and *ADAM29*_rs11946315, was selected as the best model (Table S1). The TRBA, TEBA, and CVC values of this eight-SNP model were 0.8474, 0.5377, and 10/10, respectively, after adjusting for age, gender, BMI, residence area, physical activity, education, income, smoking, and alcohol and energy intake.

### 3.4. PRSs Obtained by the Summation of Risk Alleles in the Best Model for Gastric Cancer Risk

A model containing eight SNPs was used to evaluate the association between polygenic risk scores (PRSs) and gastric cancer risk. The high-PRS group and the low-PRS group were compared, and the adjusted odds ratios (ORs) and their 95% confidence intervals (CIs) were calculated. After adjusting for the first set of covariates (covariate set 1), the adjusted OR for gastric cancer in the high-PRS group was 4.04 (95% CI: 2.68–6.11) (Table S2). This indicated that, after adjusting for other potential influencing factors, the risk of gastric cancer in the high-PRS group was more than four times that in the low-PRS group, and this association was statistically significant. In addition, the above analysis was repeated after adjusting for the second set of covariates (covariate set 2). The results showed that the adjusted OR for gastric cancer in the high-PRS group was 4.12 (95% CI: 2.71–6.27) (Table S2), which was similar to the results after adjustment for the first set of covariates. This shows that, regardless of which set of covariates was adjusted, the risk of gastric cancer in the high-PRS group was significantly higher than that in the low-PRS group. The stability of this association was further verified. Such results are of great significance for understanding the role of genetic mutation in the occurrence of gastric cancer. They help develop personalized risk assessment and intervention strategies based on genetic information. At the same time, they also emphasize the importance of considering multiple covariates when conducting genetic epidemiological studies to ensure the accuracy and reliability of the research results. These results indicated that subjects in the high-PRS group, adjusting for covariate sets 1 and 2, were at a 4.04- and 4.12-fold higher risk of gastric cancer, respectively, than subjects in the low-PRS group (*p* < 0.001). However, in covariate sets 1 and 2, no significant correlation was found between the PRS and serum total cholesterol, TG, LDL, CRP, and HDL, as well as waist circumference, hypertension, and type 2 diabetes risk (*p* > 0.05), indicating that PRSs may only be significantly associated with gastric cancer, while metabolic markers such as cholesterol and triglycerides are affected by multiple factors such as genetics, diet, and lifestyle, and the complexity and diversity of these factors may make it difficult to simply summarize the relationship between metabolic markers and gastric cancer risk. Therefore, abnormalities in metabolic markers such as serum cholesterol and triglyceride concentrations might not be directly associated with gastric cancer risk, or this association might be masked by other stronger influencing factors.

### 3.5. Interaction between the PRSs and Biochemical Parameters Influencing Gastric Cancer Risk

**Figure 2:**
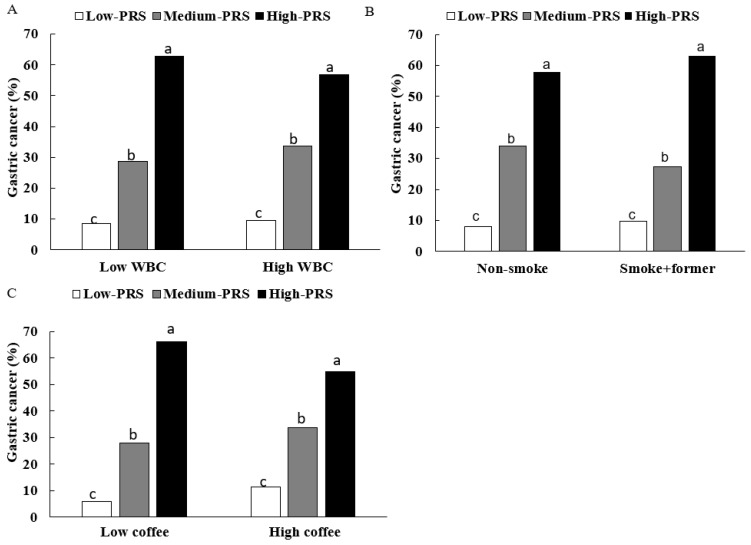
Incidence of gastric cancer according to the parameters to interact with polygenic risk scores (PRS). (**A**) Gastric cancer incidence according to their white blood cell counts (WBC, cutoff value: 4 × 10^9^/L). (**B**) Gastric cancer incidence according to their smoking status. (**C**) Gastric cancer incidence according to their coffee intake (cutoff value: 3 g/day). PRS interacted with white blood cell (WBC) counts, smoking status, and coffee intake. The participants with high-PRS were higher in the low WBC group than in the high WBC group, in the non- and former smokers than in the smokers, and in the low coffee intake (<3 cup times/week) than in the high coffee intake. *p* value indicated the interaction between PRS with designated parameters. a,b,c Different alphabets indicated significant difference among the groups at *p* < 0.05.

This study investigated the relationship between WBC count and gastric cancer risk. The risk of gastric cancer under different PRS group and WBC count combinations was analyzed. In the high-PRS group, individuals with higher WBC counts had a lower risk of gastric cancer than those with lower WBC counts (as shown in Table 4, Figure 2A, and Supplementary Figure S1A). This finding suggests that, even in people with a higher genetic risk, elevated WBC counts may have a certain protective effect on gastric cancer risk. In addition, we paid special attention to the risk of gastric cancer under the combination of a high-PRS group and a low WBC count. This study found that individuals in the high-PRS group with lower WBC counts had a 5.13-fold-increased risk of gastric cancer compared with individuals in the low-PRS group with lower WBC counts (*p* = 0.014; as shown in Table 4). This finding emphasizes that reduced WBC counts may be an important risk factor for gastric cancer in individuals with a high genetic risk. These results suggest that the WBC count may be a biomarker associated with gastric cancer risk, especially when combined with genetic information.

### 3.6. Interaction between PRSs and Lifestyle Factors Influencing Gastric Cancer Risk

The smoking and coffee intake interacted with the PRS to affect gastric cancer risk (*p* < 0.0001 and 0.04, respectively). The incidence of gastric cancer was higher in participants who smoked than in those who did not, regardless of the PRS (Figure 2B and Supplementary Figure S1B). Smokers in the high-PRS group had a higher incidence of gastric cancer than non-smokers (Table 4, Figure 2B and Supplementary Figure S1B). The smokers and non-smokers in the high-PRS group had a 3.83- and 4.29-fold higher risk of gastric cancer than those in the low-PRS group (*p* < 0.0001; Table 4). The gastric cancer incidence was higher in the high-PRS group than in the low-PRS group in participants with both a low and high coffee intake. However, the gastric cancer incidence was much higher in the participants with a high PRS and a low coffee intake (Figure 2C and Supplementary Figure S1C). The PRS was positively associated with 6.30 and 3.13 times higher risk of gastric cancer in the low and high coffee intake groups (Table 4). Those in the high-PRS group with a high coffee intake had a lower risk of gastric cancer than those with a low coffee intake (Table 4, Figure 2C, and Supplementary Figure S1C). Those in the high-PRS group with a low coffee intake had a 6.30-fold higher risk of gastric cancer than those in the low-PRS group with a low coffee intake (*p* = 0.04; Table 4). However, the rate of gastric cancer was higher in the low-coffee intake group than in the high-coffee intake group, regardless of the PRS.

### 3.7. Binding Free Energy of Food Components to Wild and Mutated Types of SEMA3C_rs1527482

The wild and mutated types of *SEMA3C_*rs1527482 had various levels of binding free energy for 20000 food components. Table 5 and Table S3 present the food components which have a low binding free energy with the wild and mutated types of *SEMA3C_*rs1527482. Some food components, including theaflavate, rugosin E, vitisifuran B, plantacyanin, and (cyanidin 3-O-beta-glucoside) (kaempferol 3-O-(2-O-beta-glucosyl-beta-glucoside)-7-O-beta-glucosiduronic acid) malonate (CK-malonate), lowered the binding energy in both wild and mutated types. Some coffee components and metabolites also contribute to a reduction in binding free energy (Table S3). However, pinotin A, delta-viniferin, sanguiin H6, and quercetin 3-O-rhamnosyl-(1->2)-rhamnosyl-(1->6)-glucoside decreased the binding energy with the wild type of *SEMA3C_*rs1527482. Withanolide B, epitheaflagallin 3-O-gallate, pomolic acid, and epigallocatechin had lower binding energies to the mutated types. *SEMA3C_*rs1527482 was positively associated with gastric cancer risk. Food components with low binding free energy may modulate and lower *SEMA3C* activity.

**Figure 3:**
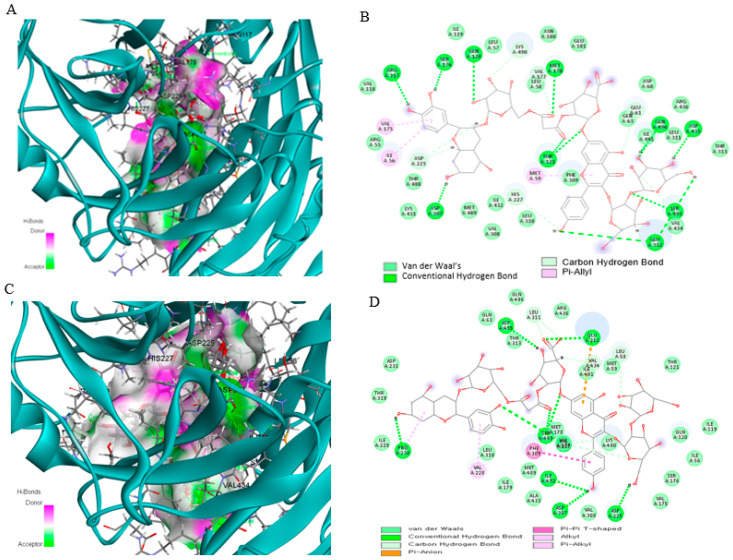
Molecular docking of C-K malonate on *SEMA3C_*rs1527482 wild (WT) and mutated types (MT). (**A**) Molecular docking of (cyanidin 3-O-beta-glucoside)(kaempferol 3-O-(2-O-beta-glucosyl-beta-glucoside)-7-O-beta-glucosiduronic acid) malonate (C-K malonate) on *SEMA3C*_rs1527482 WT. (**B**) The interaction force between C-K malonate and *SEMA3C_*rs1527482 WT. (**C**) Molecular docking of C-K malonate on *SEMA3C*_rs1527482 MT. (**D**) The interaction force between C-K malonate and *SEMA3C*_rs1527482 MT.

**Figure 4:**
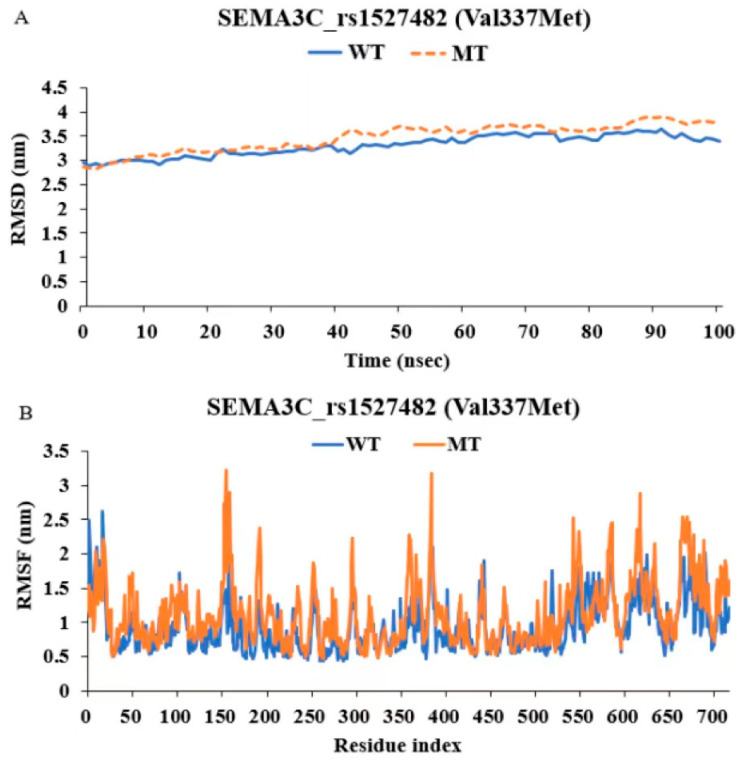
Molecular dynamic simulation (MDS) of C-K malonate on SEMA3C_rs1527482 wild (WT) and mutated types (MT). (**A**) The root-mean-square deviation (RMSD) of (cyanidin 3-O-beta-glucoside)(kaempferol 3-O-(2-O-beta-glucosyl-beta-glucoside)-7-O-beta-glucosiduronic acid) malonate (C-K malonate) on WT and MT of SEMA3C_rs1527482. (**B**) The root-mean-square fluctuation (RMSF) of C-K malonate on WT and MT of SEMA3C_rs1527482.

Through calculation and simulation, the binding free energy between the wild-type *SEMA3C* protein and the CK-malonate molecule, as well as the changes in this binding in the mutant *SEMA3C* protein, were analyzed. Figure 3A shows the binding free energy between the wild-type *SEMA3C* protein and CK-malonate through hydrogen bonding, where the pink and green parts represent the donor and acceptor of the hydrogen bond, respectively. Figure 3B provides a two-dimensional image that more intuitively shows their binding positions and intermolecular forces. The binding of CK-malonate to the mutant *SEMA3C* protein was further analyzed, as shown in Figure 3C,D. The binding energy of CK-malonate to the wild-type *SEMA3C* protein was −10.5 kcal/mol, while that to the mutant *SEMA3C* protein was −8.5 kcal/mol. This suggested that the mutation might affect the binding stability between the *SEMA3C* protein and CK-malonate. In order to more comprehensively evaluate the stability of this binding, the root mean square deviation (RMSD) and root mean square fluctuation (RMSF) of the *SEMA3C* protein (whether wild or mutant type) when bound to another molecule, CK-malonate, were also calculated. As shown in Figure 4A,B, the RMSD of wild-type *SEMA3C* protein bound to CK-malonate remained close to 3 Å throughout the simulation, indicating that their binding was relatively stable. Similarly, the RMSF of wild-type *SEMA3C* protein bound to CK-malonate also mostly remained below 3 nm, except for one exception at residue 580 in the RMSF map. These results further support the view that CK-malonate can stably bind to wild-type *SEMA3C* protein.

## 4. Discussion

In this study, we explored the effects of genetic variants on gastric cancer risk. Through a comprehensive analysis combining GWAS and GMDR, we identified 10 genetic variants significantly correlated with gastric cancer. Further analysis revealed an optimal SNP–SNP interaction model comprising eight SNPs: *DAB1*_rs7521784, *PDK1*_rs12693006, *DOCK10*_rs1045653, *MAGI2*_rs1207808, *CSMD1*_rs58499534, *MICAL2*_rs10831776, *CSNK2A1*_rs205881, and *ADAM29*_rs11946315. The SNPs demonstrated the complex genetic landscape underlying gastric cancer susceptibility. The PRSs derived from these eight SNPs demonstrated interactions between WBC count, smoking status, and coffee consumption. These findings provide novel insights into the complex interplay between genetic and environmental factors in gastric cancer risk. Our in silico analysis focused on *SEMA3C*_rs1527482, a missense mutation. We observed that specific polyphenols altered the binding affinity of this variant, suggesting its potential as a therapeutic target for gastric cancer. This discovery opens new avenues for personalized nutritional interventions in gastric cancer prevention and treatment.

The association of *DAB1*_rs7521784 with gastric cancer risk is a novel finding in our study. Previous research has identified *DAB1* mutations in Chinese patients with chronic gastritis and peritoneal metastasis of gastric cancer [29], and reduced *DAB1* mRNA expression has been observed in various cancers [30]. Similarly, our findings regarding *PDK1*_rs12693006 align with the known roles of *PDK1* in cancer-related processes and its association with poor gastric cancer prognosis, suggesting this variant’s involvement in tumor activity.

The inclusion of *DOCK10*_rs1045653 in our model is particularly interesting. DOCK proteins are known to be involved in various pathologies, including cancer, by regulating the actin cytoskeleton, cell adhesion, and migration [31]. *DOCK10*, specifically, has been shown to play roles in immune function and neuroinflammation [32]. Our study is the first to associate this genetic variant with gastric cancer risk, potentially highlighting new pathways in gastric cancer development. While the hypermethylation of *MAGI2* has been linked to gastric cancer tumorigenesis [33], our study is the first to identify an SNP in this gene associated with gastric cancer risk. This finding may provide new avenues for understanding the genetic basis of gastric cancer development.

The involvement of CUB and Sushi Multiple Domains 1(*CSMD1*)_rs58499534 in our model aligns with previous research showing the crucial roles of *CSMD1* in cancer-related processes [34]. Our study extends these findings to include a specific genetic variant associated with gastric cancer risk. Similarly, our identification of microtubule-associated monooxygenase, calponin, and LIM domain containing 2 (*MICAL2*)_rs10831776 as risk factors is consistent with previous research showing elevated *MICAL2* mRNA expression in gastric cancer tissues [35,36]. *CSNK2*, or casein kinase 2 (*CK2*), is involved in various cellular processes and has been implicated in tumor development, with *CSNK2A1* overexpression shown to promote gastric cancer progression [37]. *ADAM29* has been demonstrated to promote gastric cancer cell proliferation, migration, and invasion, with increased expression associated with poor patient survival [38]. These findings contribute to our understanding of the polygenic nature of gastric cancer risk. While each genetic variant may have a minor individual effect, their combination can significantly increase the associated risk [39].

The WBC count is a systemic inflammatory biomarker associated with an increased risk of several chronic diseases. Chronic inflammation is also known to play a role in cancer pathogenesis. A Japanese study reported that a high WBC count was a risk factor for gastric cancer in *H. pylori*-infected subjects. However, no association was observed in the *H. pylori*-negative group [40]. In this study, the incidence of gastric cancer was higher in participants with a low WBC count, and the low count of WBCs interacted with the PRS to increase the risk of gastric cancer. In the low WBC count group, individuals with a high PRS had a 5.13-fold higher risk of gastric cancer than those with a low PRS in subjects whose *H. pylori* infection status was unknown.

The International Agency for Research on Cancer (IARC) classified smoking as a carcinogen in 2004, confirming its role as a significant risk factor for gastric cancer [41]. The carcinogenic process is believed to involve gastric atrophy induced by substances such as nitrosamines and other nitroso compounds present in tobacco smoke [42]. Our study builds upon this knowledge by demonstrating an interaction between smoking status and the PRS in influencing gastric cancer risk. Notably, individuals with a high PRS who were former or current smokers exhibited a 3.83-fold-increased risk of gastric cancer compared to those with a low PRS.

Coffee’s relationship with gastric cancer is more complex and controversial. As an intricate mixture of compounds, coffee contains both potential carcinogens and anti-cancer agents. Antioxidants like phenolic compounds, diterpenes, melanoidins, and vitamin precursors may offer protective effects, while trace amounts of aromatic hydrocarbons and heterocyclic amines formed during processing could potentially promote carcinogenesis [43]. Some studies have reported a modest 7% reduction in gastric cancer risk associated with coffee consumption [44], while others have found no significant association [43]. It is important to note that the observed lower coffee consumption in the gastric cancer group may be partially attributed to dietary changes following diagnosis, rather than being solely a contributing factor to cancer development. This potential reverse causality highlights the need for prospective studies to further elucidate the relationship between coffee consumption and gastric cancer risk.

*SEMA3C*, a secreted glycoprotein of the semaphorin class 3 family, has been implicated in gastric cancer progression [45]. This protein promotes cancer growth and treatment resistance by activating signaling cascades involving the epidermal growth factor receptor (EGFR), erythroblastic oncogene B2 (ErbB2), and mesenchymal-epithelial transition (MET). These pathways are independently transactivated via plexin B1 by cognate ligands [46]. Elevated expression and activity of *SEMA3C* have been associated with increased cancer cell invasion and adhesion [45]. Additionally, *plexin B1* plays a role in modulating immune responses, which may influence cancer development. Our findings align with previous research, demonstrating that the *SEMA3C*_rs1527482 variant is positively associated with gastric cancer risk. Specifically, the minor allele of this SNP appears to confer increased susceptibility to gastric cancer. As a missense mutation, the activity of this genetic variant may be modulated by interactions with dietary components.

Molecular docking studies revealed potential interactions between small molecule food compounds and *SEMA3C*_rs1527482 (wild type and mutant), providing a quantitative metric (binding energy) for evaluating compound–protein interactions. A low binding energy means stronger interactions, which may improve or regulate protein function. Although metabolism may affect the compounds’ structure, the docking results still provide a key starting point for understanding how dietary components could have different effects based on individual genetic variations. Despite its limitations, molecular docking lays the foundation for exploring genotype-specific nutritional interventions and their impacts on health. Therefore, molecular docking research is of great significance in accelerating the development of new drugs, guiding the optimization of drug molecular structures, revealing the interaction between drugs and targets, and predicting drug metabolic pathways. It is an indispensable technology in the field of modern drug development.

An in silico analysis revealed that certain food components bind to the SEMA3C protein with binding energies below −10 kcal/mol, suggesting the potential modulation of SEMA3C activity. Interestingly, the binding affinities differed between the wild-type and mutated forms of the protein. For both variants, tea components exhibited strong binding. However, the wild-type protein showed preferential binding to components from grapes and wine, while the mutated form demonstrated stronger interactions with compounds from tea and fruit peels. These findings suggest that specific dietary elements, particularly those found in tea, grapes, and fruit peels, might differentially suppress SEMA3C activity in individuals carrying the wild-type or mutated rs1527482 allele. This potential gene–diet interaction could implicate personalized nutrition strategies in gastric cancer prevention. However, it is crucial to note that these computational predictions require validation through rigorous experimental studies.

The strengths of this study are as follows: (1) This study utilized a large sample size, ensuring strong statistical power and improving the generalizability of our findings to Korean adults. (2) We employed multiple aspects of genetic analysis, polygenic risk scores, and lifestyle to provide a more nuanced understanding of gastric cancer risk factors, thereby improving the validity and relevance of our results. (3) The interactions observed between specific food components and genetic variants provided potential practical applications. These results might help develop personalized gastric cancer prevention and management strategies. The limitations of this study are as follows: (1) The cross-sectional nature of this study limited our ability to establish temporal relationships between variables. Therefore, we could not directly infer causal relationships or track changes in disease status over time. (2) Our study population was recruited from urban hospitals, and because the study samples were mainly from urban hospitals, the results might not be applicable to a wider population in rural or remote areas. In addition, participants might have been more or less inclined to participate in the study due to factors such as health status, knowledge level, or socioeconomic status, which could also introduce selection bias. (3) The reliability of the self-reported data was often affected by environmental factors such as the memory, understanding, and honesty of the participants. In this study, gastric cancer diagnosis was self-reported and not independently verified, which might lead to inaccurate or biased information. In addition, we did not distinguish between gastric cancer subtypes because they might have different risk factors depending on the location of the tumor. (4) Patients’ lifestyles and nutrient intake were self-reported based on individual estimates of their usual intake [21]. The food intake measured by SQFFQ might not fully capture long-term dietary habits, and the collection process might be subject to bias, similar to other self-report methods. (5) *H. pylori* infection has been widely recognized as an important risk factor for gastric cancer. Failure to adjust for the confounding factor of *H. pylori* infection might exaggerate or underestimate the associations between other risk factors and gastric cancer. Such bias might affect the reliability and accuracy of the study results. Despite these limitations, our study provided valuable insights into the complex interactions between genetic and environmental factors in gastric cancer risk, laying the foundation for future research and potential prevention strategies.

## 5. Conclusions

Our study identifies a novel eight-SNP PRS model that significantly elevates the gastric cancer risk by 4.12-fold and highlights the potential role of SEMA3C_rs1527482 in gastric cancer susceptibility. We found evidence suggesting that specific components in tea, grapes, and fruit peels might differentially affect wild-type and mutated SEMA3C protein activity. Important interactions between white blood cell counts, PRSs, coffee consumption, and smoking status were revealed, amplifying the genetic susceptibility to gastric cancer and underscoring the complex interplay between genetic and environmental factors in cancer development. While these findings contribute significantly to our understanding of gastric cancer risk, it is important to acknowledge the limitations of our study, including its cross-sectional nature, reliance on self-reported food intakes and lifestyles, and lack of differentiation between gastric cancer subtypes.

Based on our results, we propose that customized nutritional plans to potentially reduce gastric cancer risk could include increasing coffee and polyphenol-rich food consumption, especially for individuals with a high PRS. Theaflavate, rugosin E, vitisifuran B, and plantacyanin could be recommended regardless of *SEMA3C*_rs1527482 variant status. Additionally, immune-boosting foods and smoking cessation strategies could be emphasized for participants with high PRSs. However, these dietary recommendations are preliminary and require further clinical validation. Future research should focus on validating these findings in larger, more diverse populations, conducting long-term clinical trials to assess the efficacy of targeted dietary interventions, and integrating other relevant genetic markers and environmental factors to develop more comprehensive and personalized prevention strategies for gastric cancer.

## Figures and Tables

**Table 1:** Socio-economic and metabolic characteristics of the participants according to gastric cancer.

	Non-Gastric Cancer (*n* = 47,994)	Gastric Cancer (*n* = 312)	Adjusted OR (95% CI)
Age (years) ^1^	53.48 ± 8.04	58.12 ± 7.85 ***	1.455 (0.987~2.145)
Genders (men: N, %)	16,808 (35.0)	168 (53.8) ***	3.369 (2.173~5.225)
Initial menstruation age ^2^	15.10 ± 1.76	15.40 ± 1.83 *	1.606 (0.771~3.345)
Menopause age ^3^	49.30 ± 4.81	49.50 ± 4.29	0.902 (0.518~1.571)
Pregnancy experience (Yes, %) ^4^	30,076 (96.6)	137 (95.8)	0.543 (0.162~1.822)
Hormone replacement therapy (Yes, %)	4963 (26.4)	28 (26.2)	0.620 (0.324~1.188)
Oral contraceptive (Yes, %)	4816 (15.5)	22 (15.4)	1.277 (0.649~2.509)
Breastfeeding (Yes, %)	25,453 (85.8)	122 (89.1)	1.234 (0.575~2.651)
Ovariectomy (Yes, %)	664 (7.5)	5 (10.4)	1.217 (0.312~4.743)
Hysterectomy (Yes, %)	3434 (11.1)	19 (13.3)	1.012 (0.519~1.976)
Body mass index (BMI, kg/m^2^) ^5^	24.00 ± 2.88	22.40 ± 3.12 ***	0.353 (0.222~0.563)
Waist circumference (cm) ^6^	80.90 ± 8.65	78.00 ± 9.04 ***	1.422 (0.636~3.179)
Plasma total cholesterol (mg/dL) ^7^	197.6 ± 35.7	186.3 ± 36.3 ***	0.492 (0.277~0.874)
Plasma triglyceride (mg/dL) ^8^	119.4 ± 64.9	99.6 ± 52.3 ***	0.606 (0.380~0.968)
Hypertension (N, %) ^9^	13,709 (28.6)	70 (22.4) *	0.757 (0.497~1.152)
Type 2 diabetes (N, %) ^10^	4256 (9.1)	28 (9.2)	0.755 (0.431~1.324)
White blood cell counts (10^9^/L) ^11^	5.73 ± 1.55	5.37 ± 1.40 ***	0.426 (0.237~0.765)
Plasma hs-CRP (mg/dL) ^12^	0.14 ± 0.36	0.15 ± 0.47	2.080 (0.937~4.615)
Education (Number, %) ^13^			
<High school	14,110 (29.7)	122 (39.2) *	
High school	20,658 (43.4)	110 (35.4)	0.602 (0.388~0.935)
College more	12,778 (26.9)	79 (25.4)	0.480 (0.301~0.764)
Income (Number, %) ^14^			
<$2000/month	13,851 (30.5)	125 (42.7) ***	
$2000–4000	27,761 (61.1)	156 (53.2)	0.803 (0.547~1.181)
>$4000	3851 (8.5)	12 (4.1)	0.288 (0.097~0.855)

The values represent means ± standard errors or number of the adults aged ≥40 (percentage of each group). The cutoff points of the reference were as follows: ^1^ <55 years old for age, ^2^ <14 years old for initial menstruation age, ^3^ <50 years old for menopause age, ^4^ non-pregnancy experience, ^5^ <25 kg/m^2^ BMI, ^6^ <90 cm for men and 85 cm for women waist circumferences, ^7^ <230 mg/dL plasma total cholesterol concentrations, ^8^ <150 mg/dL plasma triglyceride concentrations, ^9^ <140 mmHg systolic blood pressure, and <90 mmHg diastolic blood pressure plus hypertension medication, ^10^ <126 mL/dL fasting serum glucose plus diabetic drug intake, ^11^ <4 × 10^9^/L white blood cell counts, ^12^ <0.5 mg/dL serum high sensitive-C-reactive protein (hs-CRP) concentrations, ^13^ high school graduation, and ^14^ <$2000/month income. Adjusted odds ratios (ORs) are shown after adjusting for covariates, including age, gender, body mass index (BMI), residence area, physical activity, education, smoking, years with gastric cancer, and intake of alcohol and energy by logistic regression models. * Significant differences by the non-gastric cancer group at *p* < 0.05, *** *p* < 0.001.

**Table 2:** Nutrient intake and dietary patterns of the participants according to gastric cancer presence.

	Non-Gastric Cancer (*n* = 47,994)	Gastric Cancer (*n* = 312)	Adjusted OR (95% CI)
Energy intake ^1^ (%)	98.70 ± 31.5	91.80 ± 32.6 ***	0.976 (0.683~1.397)
Carbohydrate intake (En%) ^2^	71.53 ± 7.01	73.24 ± 7.16 ***	0.928 (0.552~1.561)
Protein intake (En%) ^3^	13.45 ± 2.59	13.17 ± 2.63	1.050 (0.749~1.472)
Fat intake (En%) ^4^	14.00 ± 5.43	12.63 ± 5.56 ***	0.744 (0.502~1.103)
Na intake (mg/day) ^5^	2454 ± 1389	2387 ± 1549	0.940 (0.640~1.380)
Fiber intake(g/day) ^6^	5.71 ± 2.83	5.90 ± 3.27	0.895 (0.198~4.051)
Exercise (Number, %) No Yes	21,927 (45.8) 25,932 (54.2)	121 (38.9) * 190 (61.1)	1.136 (0.801~1.612)
Smoking (Number, %) No Former smoking Smoking	34,996 (73.1) 7484 (15.6) 5383 (11.3)	185 (59.7) *** 101 (32.6) 24 (7.7)	2.715 (1.558~4.731) 0.628 (0.282~1.396)
Alcohol intake (Number, %) Mild drink (0–20 g) Moderate drink (≥20 g)	45,383 (95.2) 2291 (4.8)	307 (98.4) ** 5 (1.6)	0.181 (0.039~0.840)
Coffee intake (Number, %) ^7^ Low High	15,427 (32.4) 32,145 (67.6)	136 (43.7) *** 175 (56.3)	0.658 (0.467~0.927)
Multivitamin No Yes	43,157 (89.9) 4837 (10.1)	281 (90.1) 31 (9.9)	0.775 (0.450~1.333)
Total phenol (g/day)	2.51 ± 0.005	2.52 ± 0.041	1.204 (0.999~1.451)
Dietary inflammatory index	−19.9 ± 0.067	−21.5 ± 0.56 **	0.857 (0.716~1.026)
Fried food (Number, %) ^8^			
Low	45,184 (94.8)	300 (96.5)	
High	2481 (5.2)	11 (3.5)	1.647 (0.645~4.210)

The values represent means ± standard errors or number of the adults aged ≥40 (percentage of each group). Adjusted odds ratios (ORs) are shown after adjusting for covariates, including age, gender, BMI, residence area, physical activity, education, smoking, years with gastric cancer, and intake of alcohol and energy by logistic regression models. The cutoff points of the reference were as follows: ^1^ <estimated energy intake, ^2^ <65 energy % carbohydrate intake, ^3^ <13 energy % protein intake, ^4^ <20 energy % fat intake, ^5^ <1600 sodium intake, ^6^ <14 fiber intake, ^7^ <3 g/day coffee drinking, and ^8^ <1 time/week fried food. En%, energy percent. * Significant differences by the non-gastric cancer group at *p* < 0.05, ** at *p* < 0.01, *** *p* < 0.001.

**Table 3:** The characteristics of the 10 genetic variants of genes in gastric cancer used for the generalized multifactor dimensionality reduction analysis in adults aged >40.

Chr ^1^	SNP ^2^	Position	Mi ^3^	Ma ^4^	OR ^5^	^6^ *p* Value Adjusted	^7^ MAF	^8^ *p* Value for HWE	Gene	Functional Consequence
1	rs7521784	58175325	A	G	1.38	3.99 × 10^−4^	0.4178	0.7795	*DAB1*	Upstream of transcript
2	rs12693006	173467213	C	T	1.59	1.70 × 10^−6^	0.2374	0.6649	*PDK1*	3′ UTR
2	rs1045653	225630435	T	C	0.63	1.90 × 10^−5^	0.3389	0.2458	*DOCK10*	3′ UTR
3	rs9835646	114148557	A	C	0.61	4.44 × 10^−5^	0.196	0.4959	*ZBTB20*	Upstream of transcript
3	rs630760	124149174	G	A	1.48	2.53 × 10^−4^	0.1762	0.3554	*KALRN*	Downstream of transcript
4	rs11946315	175870844	C	T	0.69	4.59 × 10^−4^	0.2759	0.2781	*ADAM29*	Intron
7	rs1207808	78496427	C	G	0.66	2.28 × 10^−4^	0.2762	0.3319	*MAGI2*	Upstream of transcript
7	rs1527482	80427530	T	C	1.93	2.60 × 10^−5^	0.055	0.2334	*SEMA3C*	Missense
8	rs58499534	3471561	G	A	1.58	3.85 × 10^−6^	0.2156	0.2413	*CSMD1*	Upstream of transcript
11	rs10831776	12297403	G	A	0.68	3.46 × 10^−4^	0.2622	0.5937	*MICAL2*	Intron
20	rs205881	486771	T	C	1.47	1.03 × 10^−4^	0.2407	0.4981	*CSNK2A1*	Intron

^1^ Chromosome; ^2^ single-nucleotide polymorphism; ^3^ minor allele; ^4^ major allele; ^5^ odds ratio; ^6^ *p*-value for OR after adjusting for age, gender, body mass index, residence area, physical activity, education, smoking, and intake of alcohol and energy; ^7^ minor allele frequency; ^8^ Hardy–Weinberg equilibrium.

**Table 4:** Adjusted odds ratios (ORs) for the risk of gastric cancer by polygenetic risk scores (PRSs) of the best model after covariate adjustments according to low- and high-lifestyle factors.

	Low-PRS (*n* = 10,166)	Medium-PRS (*n* = 20,168)	High-PRS (*n* = 17,972)	Gene-Nutrient Interaction *p* Value
Low WBC ^1^ High WBC	1	2.355(0.604~9.181) 1.780(1.020~3.105)	5.126(1.415~18.567) 3.506(2.063~5.959)	0.014
Low energy ^2^ High energy	1	1.661(1.000~2.760) 2.709(1.044~7.033)	3.400(2.104~5.493) 7.355(2.956~18.302)	0.244
Low CHO ^3^ High CHO	1	2.004(0.421~9.529) 1.837(1.154~2.923)	7.552(1.769~32.236) 3.817(2.456~5.931)	0.298
Low protein ^4^ High protein	1	1.790(0.962~3.329) 1.909(1.007~3.622)	4.200(2.340~7.538) 4.097(2.231~7.525)	0.945
Low fat ^5^ High fat	1	1.830(1.083~3.091) 1.929(0.830~4.484)	4.294(2.617~7.044) 3.829(1.716~8.543)	0.400
No exercise Exercise	1	1.453(0.748~2.822) 2.212(1.207~4.054)	3.195(1.718~5.939) 4.985(2.799~8.878)	0.795
Non-smoke Smoke + former	1	2.208(1.232~3.957) 1.376(0.685~2.763)	4.295(2.453~7.521) 3.825(2.019~7.249)	*p* < 0.0001
Low Coffee ^6^ High Coffee	1	2.299(1.065~4.964) 1.669(0.964~2.889)	6.301(3.039~13.07) 3.129(1.858~5.267)	0.04

Values represent odds ratios and 95% confidence intervals of the adults aged ≥40. PRSs with eight SNPs were divided into three categories (1–6, 7–8, and ≥9) by tertiles as the low, medium, and high groups of the best model of GMDR. The cutoff point was as follows: ^1^ <4 × 10^9^/L white blood cell (WBC) counts, ^2^ <estimated energy intake, ^3^ <65% carbohydrate (CHO) intake, ^4^ <13% protein intake, ^5^ <20% fat intake, and ^6^ <3 g/day coffee drinking. Values represent adjusted odds ratios and 95% confidence intervals. Covariates included age, gender, BMI, residence area, physical activity, education, smoking, years with gastric cancer, and intake of alcohol and energy. The reference was the low-PRS group.

**Table 5:** Binding energy between the wild (WT, Val337) and mutated type (MT, 337Met) *SEMA3C_*rs1527482 and food components.

Both of WT and MT		
Natural compounds	Binding energy (kcal/mol)	Foods containing the selected natural compound
Trisjuglone	−11.1	Juglans regia (walnut) roots.
Rugosin E	−11.8	Cloves
Theaflavate B	−11.3	Black tea (*Camellia sinensis*).
Theaflavate A	−11.4	Black tea (*Camellia sinensis*).
Theaflavin 3′-gallate	−11.3	Black tea and commercial oolong tea
Lettowianthine	−11.7	*Annona glabra* (pond apple).
Vitisifuran B	−11.8	wine grape, *Vitis vinifera* ‘Kyohou’
Tragopogonsaponin J	−11.3	*Tragopogon porrifolius* (salsify), green vegetables
Mongolicain A	−11.1	Guava
Plantacyanin	−12.5	Cucumber, green vegetables.
WT only		
Natural compounds	Binding energy (kcal/mol)	Foods containing the selected natural compound
Pinotin A	−10.3	Red wine, including Pinotage (CCD)
Quercetin 3-O-rhamnosyl-(1->2)-rhamnosyl-(1->6)-glucoside	−10.2	Common sage, common thyme, Italian oregano, and rosemary
delta-Viniferin	−10.1	Stressed grapevine (*Vitis vinifera*) leaves
Murrayenol	−10.4	Roots of *Murraya koenigii* (curry leaf tree).
Sanguiin H6	−10.1	*Sanguisorba officinalis* (burnet bloodwort), blackberry, and red raspberry.
Isovitexin 6″-rhamnoside	−10.0	Grape and mung bean.
C-K malonate	−10.5	Chives
MT only		
Natural compounds	Binding energy (kcal/mol)	Foods containing the selected natural compound
Withanolide B	−10.6	Leaves of *Lycium chinense* (Chinese boxthorn)
Epitheaflagallin 3-O-gallate	−10.6	Black tea.
Pomolic acid	−10.8	Apple peel, rosemary, lemon balm, pomes, and spearmint.
19-Dehydroursolic acid	−10.6	*Sanguisorba officinalis* (burnet bloodwort).
Ganosporelactone B	−10.9	Spores of *Ganoderma lucidum* (reishi).
alpha-Amyrone	−10.9	*Sambucus nigra* (elderberry)
3,3′-Bisanigorufone	−11.5	Rhizomes of *Musa acuminata* (dwarf banana)
Epigallocatechin-(4 beta->6)-epicatechin 3,3′-digallate	−11.4	Oolong tea, *Camellia sinensis*
Artomunoxanthentrione epoxide	−10.8	Root bark of *Artocarpus communis* (breadfruit).
Khelmarin D	−10.7	*Citrus paradisi* and *Citrus tangerina* (Rutaceae).

Food components to lower binding energy with WT and MT *SEMA3C* rs1527482 and foods containing the selected food component. C-K malonate: (cyanidin 3-O-beta-glucoside) (kaempferol 3-O-(2-O-beta-glucosyl-beta-glucoside)-7-O-beta-glucosiduronic acid) malonate.

## Data Availability

The data were deposited in the Korean biobank (Osong, Republic of Korea).

## Supplementary Materials

The following supporting information can be downloaded at: https://www.mdpi.com/article/10.3390/nu16193263/s1, Table S1: Generalized multifactor dimensionality reduction (GMDR) results of multi-locus interaction with genes in gastric cancer risk. Table S2: Odds ratios for gastric cancer risk-adjusted for alleles of GMDR after adjustment for covariates. Table S3: Biding energy between the wild (WT, Val337) and mutated type (MT, 337Met) of SEMA3C based on rs1527482 and coffee components and its metabolites. Figure S1: Interaction between PRSs and lifestyle factors influencing gastric cancer incidence.

## Abbreviations

GC, gastric cancer group; N-GC, non-gastric cancer group; SNP, single nucleotide polymorphism; GWAS, genome-wide association study; GMDR, generalized multifactor dimensionality reduction; PRS, polygenic risk score; OR, odds ratio; *CDH1*, cadherin 1; *TP53*, tumor protein 53; *H. pylori*, *Helicobacter pylori*, *IL1B*, interleukin-1 beta; *IL1RN*, interleukin-1 receptor antagonist protein; *TNF-α*, tumor necrosis factor-α; *CYP2E1*, cytochrome P450 family 2 subfamily E member 1; *GSTM1*, glutathione S-transferase mu 1; *MTHFR*, methylenetetrahydrofolate reductase; *XRCC1*, X-ray repair cross-complementing group 1; *PRKAA1*, protein kinase AMP-activated catalytic subunit alpha 1; *MUC1*, mucin 1; *PLCE1*, phospholipase C epsilon 1; *PSCA*, prostate stem cell antigen; KoGES, Korean genomic epidemiology survey; KDCA, Korea Disease Control and Prevention Agency; BMI, body mass index; HDL-C, high-density lipoprotein cholesterol; WBC, white blood cell; SQFFQ, semiquantitative food frequency questionnaire; HWE, Hardy–Weinberg Equilibrium; LD, linkage disequilibrium; TRBA, trained balanced accuracy; TEBA, test balance accuracy; CVC, cross-validation consistency; *SEMA3C*, semaphorin-3C; PDB, protein data bank; PDBQT, protein data bank, partial charge (Q), and atom type (T); CHARMM, Chemistry at Harvard Macromolecular Mechanics; RMSD, root mean square deviation; RMSF, root mean square fluctuations; ANOVA, one-way analysis of variance; *DAB1*, disabled-1, *PDK1*, pyruvate dehydrogenase kinase-1, *DOCK10*, dedicator-of-cytokinesis-10; *ZBTB20*, zinc finger and BTB domain 20; *KALRN*, Kalirin RhoGEF Kinase; *ADAM29*, disintegrin and metalloprotease 29; *MAGI2*, membrane-associated guanylate kinase 2; *CSMD1*, cub sushi multiple domains-1; *MICAL2*, molecule interacting with CasL-2; *CSNK2A1*, casein kinase IIA1; MAF, minor allele frequency.
