# Supplementary material for: High Polygenic Risk Scores Positively Associated with Gastric Cancer Risk Interact with Coffee and Polyphenol Intake and Smoking Status in Korean Adults
Source: Nutrients. 2024 Sep 27;16(19):3263. doi: 10.3390/nu16193263 (PMC11478441; doi:10.3390/nu16193263)
Supplement: Supplementary file 1 [file nutrients-16-03263-s001.zip › nutrients-3178428-supplementary.pdf]

Table S1 Generalized multifactor dimensionality reduction (GMDR) results of multi-locus interaction with genes in gastric cancer risk

		No Adjustment				Adjusted for age, gender, BMI, residence area, physical activity, education, smoking, intake of alcohol and energy			
Model Number	SNP	TRBA	TEBA	P value	CVC	TRBA	TEBA	P value	CVC
Model 1	*CSMD1*_rs58499534	0.5641	0.5509	10 (0.0010)	8/10	0.5607	0.5479	10 (0.0010)	8/10
Model 2	*PDK1*_rs12693006 *MAGI2*_rs1207808	0.5826	0.5419	7 (0.1719)	4/10	0.5770	0.5400	8 (0.0547)	3/10
Model 3	*DAB1*_rs7521784 *PDK1*_rs12693006 *CSMD1*_rs58499534	0.6107	0.5520	9 (0.0107)	4/10	0.6037	0.5463	9 (0.0107)	4/10
Model 4	Model 2 plus *CSMD1*_rs58499534 *CSNK2A1*_rs205881	0.6427	0.5617	10 (0.0010)	5/10	0.6343	0.5572	10 (0.0010)	5/10
Model 5	Model 3 plus *MAGI2*_rs1207808 *CSNK2A1*_rs205881	0.6843	0.5716	10 (0.0010)	8/10	0.6728	0.5650	10 (0.0010)	9/10
Model 6	Model 3 plus *DOCK10*_rs1045653 *MAGI2*_rs1207808 *ADAM29*_rs11946315	0.7399	0.5419	9 (0.0107)	9/10	0.7240	0.5235	8 (0.0547)	6/10
Model 7	Model 6 plus *CSNK2A1*_rs205881	0.8063	0.5108	8 (0.0547)	6/10	0.7870	0.5073	8 (0.0547)	5/10
Model 8	Model 7 plus *MICAL2*_rs10831776	0.8706	0.5419	10 (0.0010)	10/10	0.8474	0.5377	10 (0.0010)	10/10
Model 9	Model 8 plus *KALRN*_rs630760	0.9228	0.5349	10 (0.0010)	10/10	0.8962	0.5372	9 (0.0107)	10/10
Model 10	Model 9 plus *ZBTB20*_rs9835646	0.9530	0.5268	9 (0.0107)	10/10	0.9262	0.5312	9 (0.0107)	10/10

TRBA, trained balanced accuracy; TEBA, test balance accuracy; CVC, cross-validation Consistency; sign test, result and P value for the significance of GMDR model by sign test with and without adjusting for covariates designated in the table; BMI, body mass index.

Table S2. Odds ratios for gastric cancer risk adjusted for alleles of GMDR after adjustment for covariates

	Covariate set 1			Covariate set 2	
	Low- PRS (n=10,166) F: n= 6,626 M: n= 3,540	Medium-PRS (n=20,168) F: n= 13,099 M: n= 7,069	High-PRS (n=17,972) F: n= 11,605 M: n= 6,367	Medium-PRS (n=20,168) F: n= 13,099 M: n= 7,069	High-PRS (n=17,972) F: n= 11,605 M: n= 6,367
Gastric cancer	1	1.784 (1.151～2.763)*	4.042 (2.675～6.105)***	1.855 (1.189～2.893)**	4.117 (2.705～6.268)***
Serum Total cholesterol (mg/dl)	1	0.950 (0.891～1.012)	0.970 (0.909～1.035)	0.949 (0.890～1.013)	0.968 (0.907～1.034)
Serum TG (mg/dl)	1	1.035 (0.977～1.097)	1.032 (0.973～1.095)	1.038 (0.979～1.101)	1.036 (0.976～1.100)
Serum LDL (mg/dl)	1	0.929 (0.858～1.006)	0.971 (0.896～1.052)	0.930 (0.858～1.008)	0.974 (0.898～1.056)
Serum CRP (mg/dL)	1	1.000 (0.876～1.142)	0.984 (0.859～1.126)	1.015 (0.887～1.161)	1.001 (0.873～1.149)
Serum HDL (mg/dl)	1	1.040 (0.985～1.099)	1.047 (0.990～1.108)	1.055 (0.998～1.116)	1.055 (0.996～1.117)
Waist circumference	1	0.959 (0.889～1.034)	0.953 (0.882～1.029)	0.970 (0.899～1.048)	0.963 (0.890～1.041)
Hypertension	1	1.042 (0.983～1.104)	1.018 (0.960～1.081)	1.036 (0.976～1.099)	1.020 (0.961～1.083)
Type 2 diabetes	1	0.968 (0.888～1.056)	0.938 (0.859～1.025)	0.972 (0.890～1.062)	0.937 (0.856～1.025)

Values represent odds ratios and 95% confidence intervals in the adults aged ≥40. F: female; M: male; TG: triglyceride; LDL: low-density lipoprotein; CRP: C-reactive protein; HDL: high-density lipoprotein; The polygenic risk scores (PRS) of the 8 SNP model generated from GMDR was divided into three categories (1-6, 7-8, and ≥9) by tertiles as the low PRS, medium PRS, and high PRS, respectively. The low PRS was the reference for both Covariate set 1 and Covariate set 2. Covariate set 1: adjusted for age, gender, body mass index (BMI), and residence area. Covariate set 2: adjusted for age, gender, BMI, residence area, physical activity, education, smoking, years with gastric cancer, and intake of alcohol and energy. Significantly different from the major allele in logistic regression analysis after adjusting for covariates at * $P < 0.05$, ** $P < 0.01$, ***$P < 0.001$.

Table S3. Biding energy between the wild (WT, Val337) and mutated type (MT, 337Met) of SEMA3C based on rs1527482 and coffee components and its metabolites.

Natural compounds	Binding energy (kcal/mol)	
	Wild type	Mutant type
Coffee ingredients		
Caffeic acid ethyl ester	-6.4	-6.4
Hydroxycaffeic acid	-6.2	-7
(-)-Quinic acid	-6.3	-6.3
1,5-Dicaffeoylquinic acid	-7.9	-7.9
Malvidin 3-(6"-p-caffeyglucoside)	-8.6	-8.6
Caffeine	-6.1	-6.1
Caffeic acid	-6.5	-6.5
trans-Caffeic acid	-6.5	-6.2
Cryptochlorogenic acid	-7.7	-7.7
Neochlorogenic acid	-8.1	-8.1
Isoferulic acid	-6.6	-6.4
Caffeic acid 3-glucoside	-8.1	-8
Malvidin 3-chlorogenic acid glucoside	-7.7	-7.9
3-(3,4-Dihydroxyphenyl)propanoic acid	-6.3	-6.2
trans-Caffeic acid [apiosyl-(1->6)-glucosyl] ester	-8.5	-8.6
Chicoric acid	-8.3	-7.7
Glucocaffeic acid	-6.8	-7.5
cis-Caffeic acid	-6.5	-6.5
trans-Neochlorogenic acid	-7.6	-8.1
Metabolites of coffee in the body		
3-(3-Hydroxyphenyl) propanoic acid	-5.9	-5.9

1,3-Dimethyluric acid	-6.8	-6.8
Paraxanthine	-6.8	-6.8
3-Methylxanthine	-6.5	-6.5
3-Methyluric acid	-7	-7
9-Methyluric acid	-6.9	-6.9
3,7-Dimethyluric acid	-7.3	-7.3
1,9-Dimethyluric acid	-6.9	-6.9
1,3-Dimethyluracil	-5.3	-5.3
1-Methyluric acid	-7.2	-7.2
7-Methylhypoxanthine	-8.9	-9
7,9-Dimethyluric acid	-7	-7
5-Acetylamino-6-amino-3-methyluracil	-7	-7
8-Chloroxanthine	-6.8	-6.9
Debrisoquine	-7.4	-7.4

Figure S1. Interaction between PRSs and lifestyle factors influencing gastric cancer risk

A. Interaction of gastric cancer incidence and PRS according to low- and high white blood cells (WBC)

B. Interaction of gastric cancer incidence and PRS according to smoking status

C. Interaction of gastric cancer incidence and PRS according to coffee consumption

A

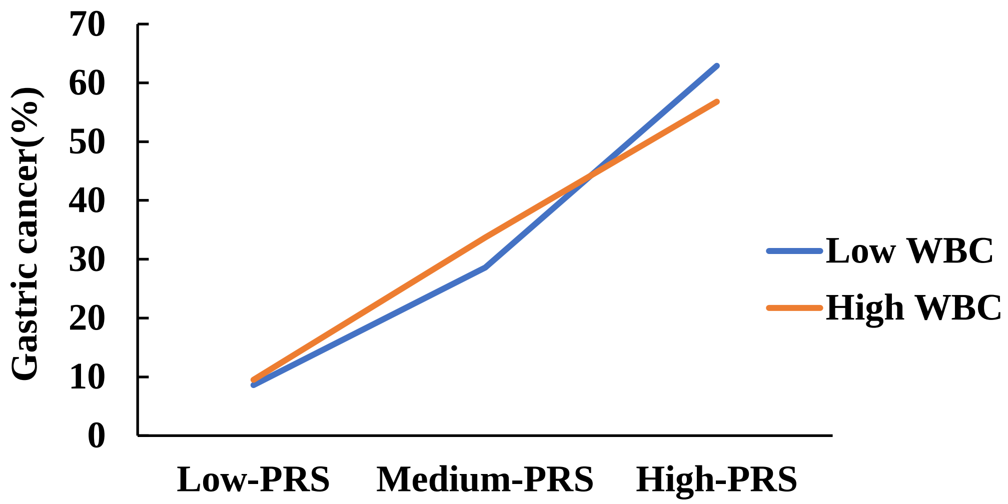

B

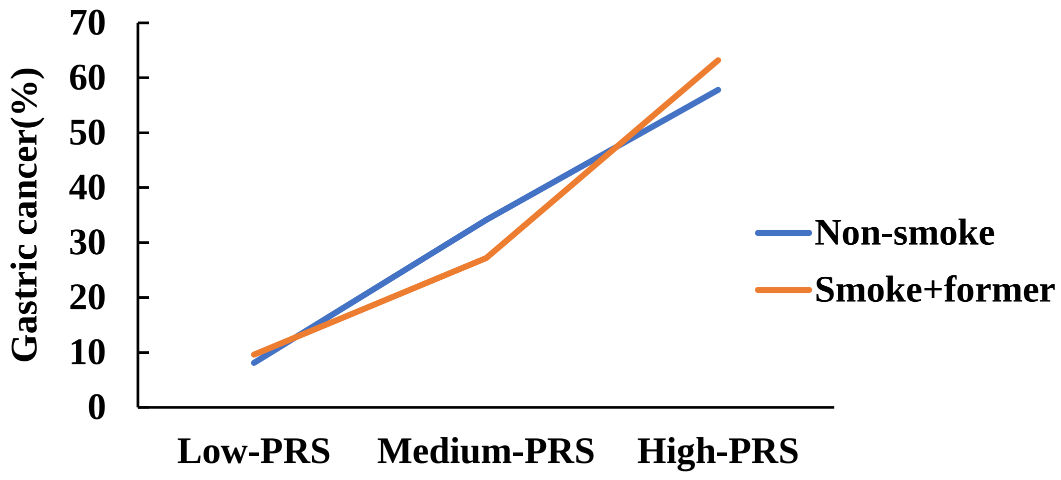

C

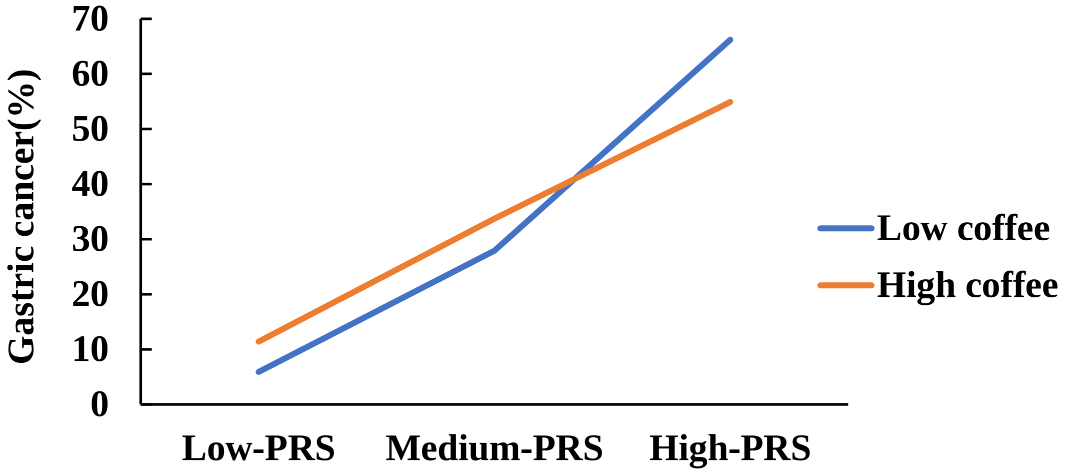
Gastric cancer(%)
70
60
50
40
30
20
10
0
Low-PRS
Medium-PRS
High-PRS
Low coffee
High coffee
